# Intention to use a mobile phone to receive mental health support and its predicting factors among women attending antenatal care at public health facilities in Ambo town, West Shoa zone, Ethiopia 2022

**DOI:** 10.1186/s12913-023-10392-z

**Published:** 2023-12-06

**Authors:** Wabi Temesgen Atinafu, Kefyalew Naniye Tilahun, Tesfahun Melese Yilma, Zeleke Abebaw Mekonnen, Agmasie Damtew Walle, Jibril Bashir Adem

**Affiliations:** 1 Department of Public Health, College of Medicine and Health Sciences, Ambo University, Ambo, Ethiopia; 2 Department of Health Informatics, Institute of Public Health, University of Gondar, Gondar, Ethiopia; 3 Department of Health Informatics, College of Health Sciences, Mattu University, Metu, Ethiopia; 4 Department of Public Health, College of Medicine and Health Sciences, Arsi University, Asella, Ethiopia

**Keywords:** mHealth, ANC, TAM, Intention to Use, Mental Health, Ethiopia

## Abstract

**Background:**

Mental health problems are the most common morbidities of women during the prenatal period. In LMICs mobile phones have been identified as a good vehicle for monitoring individuals with a high risk of mental health conditions. However, evidence is scarce and the purpose of this study was to assess the intention to use a mobile phone to receive mental health support and its predicting factors among women attending antenatal care at public health facilities in Ambo town, Ethiopia 2022.

**Methods and materials:**

An institutional-based cross-sectional study design was conducted from May 20^th^ to June 20^th^, 2022. A total of 715 prenatal women were included and a systematic random sampling technique was employed. An interviewer-administered structured questionnaire was used. Collected data was exported to SPSS version 25 for the descriptive part, and AMOS 26 structural equation modeling was also used to describe and assess the degree and significance of relationships between variables.

**Results:**

A total of 699 (97.8% response rate) responded to complete all the questionnaires. About 530 (77.3%) 95% CI (74%-80.3%) of women intended to use a mobile phone to receive mental health support. The perceived usefulness has a positive effect on attitude (β = 0.391, *p* < 0.001) and intention to use (β = 0.253, *p* < 0.001). The perceived ease of use influences perceived usefulness (β = 0.253, *p* < 0.001) and attitude β = 0.579, *p* < 0.001). The intention to use is positively affected by attitude (β = 0.662, *p* < 0.001).Trust has a positive effect on perceived usefulness (β = 0.580, *p* < 0.001) and intention to use (β = 0.113, *p* = 0.005). Subjective norm has a direct positive effect on perceived usefulness (β = 0.248, *p* < 0.001). Attitude serves as a partial mediator between perceived usefulness and intention to use and a complete mediating role between perceived ease of use and intention to use.

**Conclusion:**

The level of intention to use a mobile phone among prenatal women is relatively high and attitude, perceived usefulness, and trust had direct positive effects on intention to use a mobile phone. Therefore, hospitals and healthcare providers should take proactive measures to implement the strategies and policies for providing mobile phone-based mental health support to prenatal women in remote areas.

**Supplementary Information:**

The online version contains supplementary material available at 10.1186/s12913-023-10392-z.

## Background

Maternal health is a critical issue that needs to be addressed worldwide. Women worldwide lose their lives because of issues during pregnancy and childbirth every day due to different tragedies [1, 2]. Among these antenatal mental disorders are serious health problems and pose multiple dangers to both the mother and her fetus [2]. Globally mental health conditions are among the commonest morbidities in women during pregnancy which imposes negative health and health-related consequences for the woman, her child, and her family [3]. The World Health Organization (WHO) defines mental health as a state of well-being in which a person recognizes his or her abilities, can cope with everyday stresses, work productively and fruitfully, and contribute to his or her community [1].

The commonest mental health conditions during the pregnancy period are depression and anxiety, with nearly 12% of women experiencing depression, 13% experiencing anxiety at some point, and many women experiencing both [4, 5]. These mental health conditions can be treated in a different way using modern technology such as mobile phones. According to the current evidence available, mobile network coverage has continued to expand and mobile device penetration has reached unprecedented levels around the world [6].

Furthermore, according to the WHO, mHealth has the potential to transform healthcare delivery and cause a paradigm shift in healthcare delivery processes all over the world [7]. The World Health Organization's Global Observatory for eHealth described mHealth as any medical or public health practice that is supported by mobile devices such as mobile phones, patient monitoring devices, personal digital assistants, and other wireless devices [8].

Although there is growing evidence that mobile phones can play an important role in health care delivery, particularly mental health care, these technologies were not accepted as much as needed by different end users when tested by using different models [9]. To enhance maternal and child health outcomes, testing the acceptance or rejection of women's attitudes toward these innovative treatment options such as mobile phones is needed to expand women's access to mental health care.

According to the World Health Organization, the gap between the need for and accessibility of treatment for mental disorders in pregnancy is growing, with between 35 and 50% of mentally ill women receiving no care because appropriate treatment services are scarce [10]. Around 10% of women experience mental health conditions during pregnancy around the world, with the burden being higher in low and middle-income countries (around 15.9%), and depression being one of the most common mental disorders during pregnancy [11].

Poor maternal mental health affects more than one out of every ten women during pregnancy, and it can have a catastrophic impact on both her fetus and the woman [12]. Although mobile health has been shown to have a positive impact on clinical outcomes of mental health of pregnant women the acceptance and intention to use mobile phones for maternal healthcare are limited [13, 14]. MHealth tools like mobile phones have the potential to be scalable, cost-effective, and benefit both individual patients and the healthcare system at the same time [15–18].

In light of the Royal College of Obstetricians and Gynecologists recently published recommendations on the use of remote means to provide support to women throughout the pregnancy period, digital technologies like mobile phones may offer an innovative way to support women's mental health needs [19, 20]. Using a mobile phone to meet the demand for mental health during pregnancy is one possible solution [10].

A mobile phone-based service could be one answer to address the demand for mental health support during pregnancy. One advantage of using a mobile phone is that it saves time and money. Clinicians spend far less time with each woman than they would in traditional face-to-face support. In addition to that, there is also much worry about how pregnant women seek help for mental health issues, particularly in low and middle-income countries [12, 20]. The use of digital technologies to provide mental health information and treatment may be a valuable option; however, it is still necessary to determine the acceptability of such tools in the prenatal population, particularly in countries where these e-mental health tools have not yet been developed, such as Ethiopia.

In Africa, this high prevalence of mental illness among pregnant women highlights the need for mental health services within maternal health care systems, particularly in Sub-Saharan Africa [21]. According to research done in sub-Saharan Africa, prenatal mental disorders, with prevalence rates ranging from 8.3 to 41% [21]. Ethiopia has the highest rate of mortality during pregnancy in the world, at 33 per 1000 births [22]. More than one-fifth (23.56%) of pregnant women in Ethiopia suffer from mental health conditions [23].

Identifying necessary prerequisites before the actual implementation of the system will help to improve the implementation status. Examining pregnant women's intention to use a mobile phone is considered critical because its success depends on the intention to use it of the end users. Therefore, determining intention and predicting factors for receiving mental health support through mobile phones among prenatal women will provide input for planners and decision-makers for perinatal mental health care intervention.

### Theoretical background of the model and hypothesis

Technology Acceptance Model is one of the models that is used to investigate the intention to use information technologies [24, 25] it increases the power of predicting and explaining the TAM model when it is modified. Davis formulated the theory which is based on two previous models Ajzen and Fishbein's Theory of Reasoned Action (TRA) and Ajzen's Theory of Planned Behavior (TPB) [26] to predict and explain a person’s adoption of information technology [27].

The Technology Acceptance Model (TAM) aims to better understand why people accept or reject certain technologies, as well as how technology design might promote user acceptance [28]. TAM is nowadays used in a multitude of health-related applications and interventions [29–31], where it is now regarded as a widely accepted and practical theory of technology acceptance in the health field [32].

This study aims to identify factors that influence behavioral intention to use (BIU) a mobile phone. The extended TAM investigates some of the external variables listed in the original TAM and lays a considerably higher emphasis on the influence of social factors on a potential user's assessment of the utility of a system [33] and it modifies the TAM model by incorporating key essential variables that were recognized as relevant in the situation of intention to use a mobile phone in the health care area.

This study extended TAM in the intention to use a mobile phone among prenatal women to receive mental health support and its predictor factors domain by including three external variables, perceived trust, subjective norm, and perceived privacy, based on previous research and theoretical concepts. However, the actual use was removed from the original model since the study deals with future predictions to use the systems and the system was not in use up to the study period.

Perceive usefulness: is generally described as a person's belief that using a system will help him or her to perform better [32, 34]. A study done in Egypt [35], and the United States [32], indicated that the perceived usefulness of health support via mobile phones has a significant impact on women's attitudes toward and intention to use technology. According to research on technology acceptance in various domains, PU is the primary determinant factor for new technology acceptance [10, 36]. The proposed hypotheses are in Supplementary material 1 (H1 and H2).

Perceived ease of use: the degree to which a person believes that a particular technology will be simple and easy to use [36–38]. A study done in China [39], indicates that PEOU has a significant effect on perceived usefulness. Studies done on the Intention to use e-health in Ethiopia [37], Taiwan [40], and Malaysia [41] showed that perceived ease of use has a direct positive effect on the intention to use, Perceived usefulness, and attitudes toward using mobile phone services. The proposed hypotheses are in Supplementary material 1 (H, H4 and H5).

Attitude: Individual's positive or negative feelings about performing the target behavior [42]. A study conducted in Turkey regarding the intention to use mobile health [42], discovered that a person's intention to use mobile health was influenced by their attitude. For the proposed hypothesis see Supplementary material 1 (H6).

Subjective norm: is defined as a person's perception that a significant proportion of people important to him/her think he/she should or should not perform the behavior [43]. Studies, conducted on the intention to use mobile health for mental health in India [44], Saudi Arabia [33], and China [45], indicate that subjective norms have a direct positive effect on perceived usefulness and intention to use. The proposed hypotheses are in Supplementary material 1 (H7 and H8).

Perceived trust: A willingness to rely on an exchange partner in whom one has confidence [46]. Trust is a factor that explains a person's level of assurance that utilizing m-health applications is safe and that there is no threat to one's privacy [47].

The studies done in Pakistan on the intention to use telemedicine [48], China on the intention to use mobile health [49], and Bangladesh on the intention to use a smartphone [50] indicate that trust had a direct positive effect on perceived usefulness and intention to use.

As a result, this study expands on the original TAM by including trust in the intention to use mobile phone services. The proposed hypotheses are in Supplementary material 1 (H9 and H10).

Perceived privacy: is defined as the state of being alone and it also refers to an individual's or group's right to isolate oneself or information about themselves [51, 52]. The extent to which a client believes he or she has the right to regulate the acquisition and use of personal data, even after it has been exposed to others [48].

Studies conducted in Pakistan on telemedicine [48], in China on the intention to use telehealth [53], Germany on privacy concerns on intention to use PHR [54], indicated that Perceived Privacy had a positive effect on the intention to use. The proposed hypothesis is in Supplementary material 1 (H11).

Mediation effects: The mediators act as a bridge to pass the effects of latent variables to dependent variables [53]. According to studies, Attitude is a mediator of the effects of PU on BI [55]. TAM constructs such as PU, and attitude play an important role as mediators in the model to explain user IU [56]. However, to the best of our knowledge, there is no empirical study that tests the mediating effect in the relationship between SN, PU, PEOU, ATT, and intention to use a mobile phone to receive mental health support among pregnant women. The proposed hypothesis see in Supplementary material 1 (H12, H13, H14, H15 an H16).

**Fig. 1:**
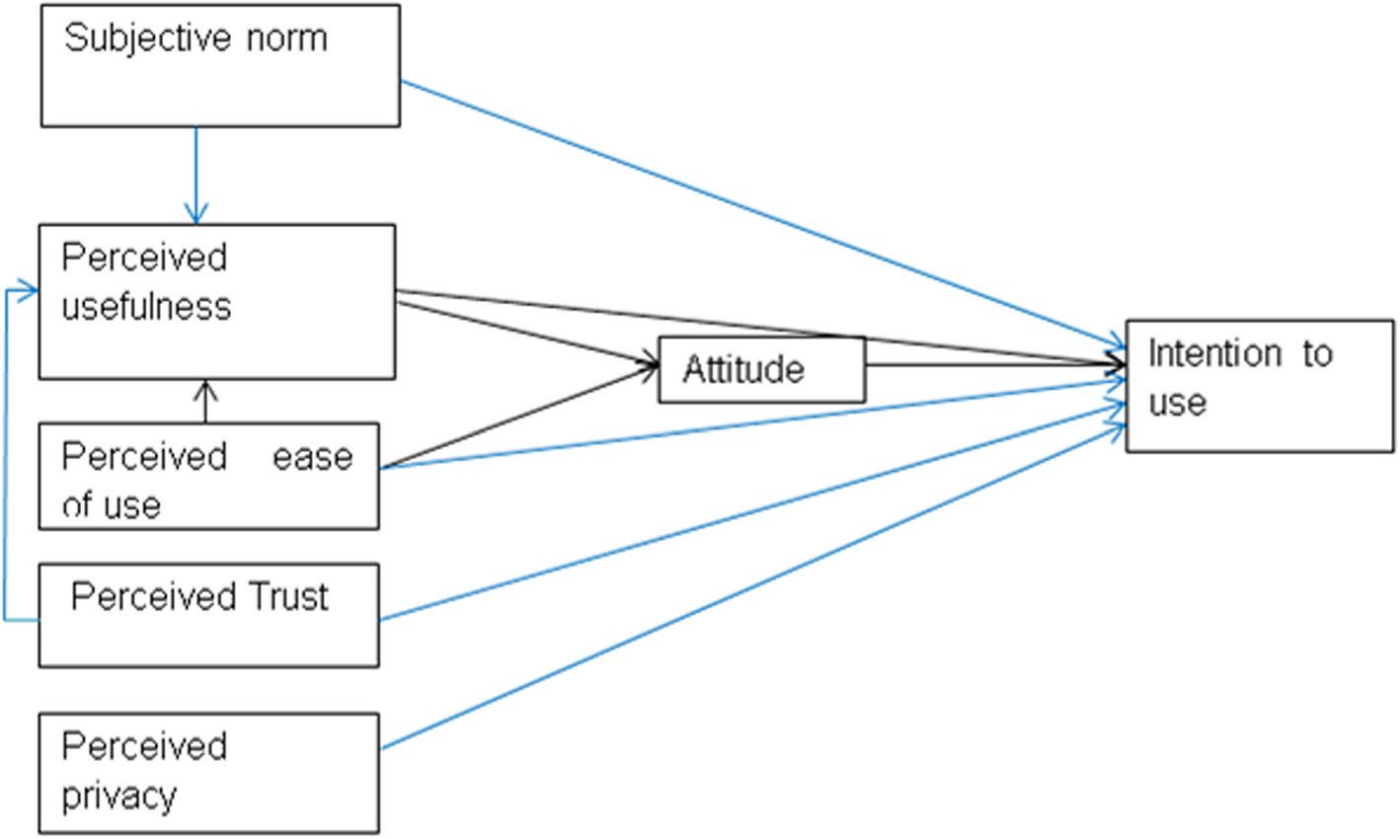
The original model (the black line), and the modification proposed model in this study (the blue line) [34]

The proposed research variables, their relationships, the research framework, and our hypotheses are explained in Fig. 1.

## Methods

### Study design and setting

An institutional-based cross-sectional study design was conducted from May to June 2022 at Ambo town public health facilities, in West Shoa, Ethiopia. Ambo town is located in the West Shoa zone of the Oromia region 110 km away from, Addis Ababa (capital city of Ethiopia). According to the health bureau of Ambo town, the total population of the town is 80,712 with 39,553 males and 41,159 females. The number of prenatal women who have followed up at public health facilities in Ambo town is estimated to be 1486. There are two hospitals (Ambo General Hospital and Ambo University Referral Hospital), and two health centers (Ambo health center and Awaro health center).

### Study participants and sample size determination

In this study, all pregnant women attending antenatal care at public health facilities in Ambo town during the study period were taken as the study population.

The minimum sample size is determined based on the number of free parameters in the hypothetical model and considering the one to ten (1:10) ratio of respondents to free parameters to be estimated has been recommended [57]. This means for any free parameter in the hypothesized model there should be 10 respondents. Accordingly, considering the 65 parameters to be estimated based on the hypothesized model and taking participants to a free parameter ratio of 10, the minimum sample required is 650. The sample size calculated accounts for the non-response rate of 10%, therefore the final sample size becomes 715.

### Sampling procedures

A systematic random sampling technique was used to select the study participants. The study participants were selected from all public health facilities in Ambo town. The total number of prenatal women who visited the health facilities within the time of the data collection period was calculated based on the health facilities' previous monthly report and then the calculated sample size was distributed across each public health facility proportionally to the size of the prenatal women at each health facility. The first respondent was selected randomly and then followed at every interval.

### Data collection tools and procedure

Data was collected by mobile-based KoboCollect version 2022.1.2. KoboCollect is a platform for open-source data collection created to assist organizations and academic institutions. The interviewer-administered, pretested structured questionnaire was utilized. For the latent variables, a standard questionnaire was adapted from different literature [34, 39, 43, 50, 58, 59]. The structured questionnaires were comprised of socio-demographic and intention to use mobile phone variables.

Intention to use is measured by four items, perceived usefulness is measured by four items, perceived ease of use is measured by four items, subjective norm is measured by three items, attitude is measured by three items, perceived trust is measured by three items, and perceived privacy measured by three items. The questionnaire was developed to test the hypotheses. The final questionnaires include 24 items that measure the different concepts. A 5-point Likert scale was utilized, with responses ranging from strongly disagree (1) to strongly agree (5).

### Data quality control

One day of training was given to six data collectors and two supervisors on the objective of the study, overall formats to familiarize them with the instrument and data collection procedures and data collecting tools, respondents' approach, data confidentiality, and respondents right before the data collection date.

Data collectors were BSc graduate midwives who have experience in antenatal care and supervisors were graduates of public health. The questionnaire was developed in English first, then was translated forward and backward to verify conceptual continuity (English to Afan Oromo then English) and (English to Amharic then English), and then an interview was conducted in both languages Afan Oromo and for those not understand well Afan Oromo were interviewed by Amharic for appropriateness, and ease of approaching the study participants.

Before the actual data collection, pretesting of the questionnaire was done at Gudar General Hospital which is out of the study area, and the appropriate modifications were made afterward. The Cronbach's alpha results of the pretest on the latent variables of PU (0.876), PEOU (0.798), ATT (0.967), SN (0.843), PT (0.923), PP (0.873), and ITU (0.897). Finally, the actual data for the study were collected from pregnant women attending antenatal care at public health facilities in Ambo town. The supervisors were examining the completeness and accuracy of the data every day. Proceeding to analyze the data, the data was cleaned up and cross-checked.

### Data processing and analysis

Before going for analysis the collected data was exported to SPSS version 25 for descriptive data analysis and AMOS version 26 was used to analyze the measurement model and structural model assessment. The socio-demographic data and mobile phone patterns were analyzed descriptively using SPSS and the result was presented using a frequency table. The proportion of intention to use a mobile phone to receive mental health support among pregnant women was computed descriptively and the finding was presented using a bar graph. The outcome variable intention to use was operationalized as intended or unintended based on the median score. The score above the median was declared intended to use and below was non-intended to use.

Before conducting measurement and structural model assessment, the maximum likelihood estimation method was used and the SEM assumption was checked.

Multivariate normality was checked using kurtosis and critical ratio. After we compute multivariate normality the data deviated from the normal distribution. To test multivariate normality, a kurtosis absolute value of less than 5, a critical ratio between -1.96 and + 1.96 [60, 61].

As findings showed from these results the data were not normally distributed. Due to this result, the assumption of maximum likelihood estimation is not fulfilled. Therefore, multivariate none normality was managed using the bootstrapping technique. Therefore, Maximum likelihood estimation with bootstrapping was used in this study which is an increasingly popular and promising approach in several contexts, and this resampling method can be used to correct fit and standard errors for non-normality in SEM.

The presence of Multicollinearity among independent variables was evaluated using the variance inflation factor (VIF) at a cut-off point of less than 10 and tolerance greater than 0.1 [62]. The VIF ranges from 1.031 to 1.221 in this study, and the tolerance ranges from 0.819 to 0.970 also used the correlation coefficient method to test for Multicollinearity between exogenous observed variables, and all Pearson's correlations were less than 0.8, which is the recommended value for ruling out Multicollinearity. The results of this study of all Pearson's correlations are less than 0.8. The results show that there was no Multicollinearity among independent variables. The other assumption was multiple measurements that were used to measure each latent variable in the structural equation model; three or more observed variables must be used. This assumption was fulfilled that all our unobservable variables had at least three indicators used.

### Measurement model

Before testing the hypothesis, confirmatory factor analysis (CFA) was undertaken. In measurement model assessment, reliability, validity, and Discriminant validity of items were determined using Cronbach’s alpha (α), standardized factor loading, composite reliability (CR), average variance extracted (AVE), and the square root of the AVE and the cross-loading matrix were to check for the reliability, validity and discriminant validity of constructs by using AMOS version 26. The values of Cronbach’s alpha and Composite reliability are typically accepted for a value of 0.7 or above to measure the internal consistency of the variables [63]. Convergent validity was measured by an average variance extracted (AVE) of at least 0.50, and Factor loading is considerably above 0.50 [49, 64]. The square root of the AVE and the cross-loading matrix were measured to determine discriminant validity and according to the loading and cross-loading results, the measurement items have higher loading under their latent constructs than with other constructs [65–67]. The model’s overall goodness of fit was measured and assessed based on standards from previous studies [37] using Chi-square ratio(x^2^/df) (< 3), normal fit index (NFI > 0.9), the goodness of fit index (GFI > 0.8), adjusted goodness of fit index (AGFI > 0.8), comparative fit index(CFI > 0.9), root mean square of standardized residual (RMSR < 0.08), and Root Mean Square Error of Approximation (RMSEA < 0.08) [49, 68].

### Structural model

Following the evaluation of the measurement model, the structural model assessment was performed to build a structural equation model for influencing factors of pregnant women’s behavioral intention to use a mobile phone to receive mental health support. There were 7 latent variables and 24 observed variables in this structural equation model. AMOS version 26 was further applied to test the structural equation model fitting and hypothesis test of influencing factors of pregnant women’s behavioral intention to use a mobile phone. The structural model fit statistics suggested a satisfactory model fit: GFI = 0.938, AGFI = 0.862, NFI = 0.945, CFI = 0.958, SMSR = 0.0346, RMSEA = 0.066 and x^2^/df = 2.317. The maximum likelihood estimation method was used. Squared multiple correlations (R^2^), were declared as representing the proportion of variance in the endogenous constructs which can be explained by the predictors.

To test the proposed hypothesis the standardized regression weights that show the strength of association between constructs [69], path analysis, and the *p*-value with a cutoff point $<0.05$ were considered. The bootstrap method to test the mediation effects was used to analyze the relationship between latent variables [53, 70]. To determine the total, direct, and indirect effects of the variables, we conducted a bootstrap analysis and bootstrap bias-corrected samples [71, 72]. Statistical significance was regarded to be shown by values of *P* < 0.05 (two-tailed) [73].

### Consent to participation and ethical clearance

Study participants provided written informed consent. For those who were able to read and write, they read and signed the consent form. For those who were unable to read and write, the informed consent was read aloud to them by the interviewer. If they agreed, they indicated their agreement with a finger sign. After obtaining informed consent, the interviewer proceeded to read out the questions. The study protocol was evaluated and approved by the University of Gondar's ethical review board, following the Declaration of Helsinki guidelines. Additionally, each public health facility provided a letter of authorization. Data gathering tools did not contain participant names or other personal information.

## Results

### Socio-demographic characteristics of respondents

A total of 715 pregnant women at each public health facility in Ambo town were approached, 699 of them being informed consented, and responded to complete all the questionnaires at public health facilities with a 97.8% response rate. The age of the respondents ranged from 18–49 years with a median age of 29 with an IQR of 9 years.

As shown in Table 2, 402 (57.5%) women belonged to an age group of 25–34 years. The majority of them were married 393(95%) and Orthodox Christians 311(44.5%) followed by protestant Christians 291(41.6%) by religion. From the total (*n* = 699) respondents 543(77.7%) were urban resident. Two hundred eighty-eight (40.5%) were housewives and 500(71.6%) of respondents had at least secondary education (Table 1).

**Table 1:** Socio-demographic characteristics of pregnant women attending ANC at public health facilities in Ambo town west Shoa zone, Ethiopia, 2022G.C (*n* = 699)

Characteristics	Total (%)
Age	
< = 24	157 (22.5%)
25–34	402 (57.5%)
> = 35	140(20.0%)
Marital status	
Currently married	664(95%)
Currently not married	35(5%)
Religion	
Orthodox	311(44.5)
Muslim	74(10.6%)
Protestant	291(41.6%)
Others^1^	23(3.3%)
Educational background	
Unable to read and write	63(9%)
Able to read and write but no formal learning	34(4.9%)
Primary level (1–8)	102(14.6)
Secondary level (9–12)	186(26.6%)
Diploma (I-IV)	164(23.5%)
Tertiary and above	150(21.5%)
Occupation	
Student	18(2.6)
Employed	197(28.2%)
Merchant	81(11.6%)
Farmer	82(11.7%)
Housewife	283(40.5%)
Others^2^	38(4.5%)
Residence	
Urban	543(77.7%)
Rural	156(22.3%)
Distance to health facilities in minutes	
= < 15	342(48.9)
16–30	243(34.8)
> 30	114(16.3)
Number of childbirths	
No child	169(24.2%)
One-two	378(54.1%)
Three-four	123(17.6%)
Greater than four	29(4.1%)
ANC visit number	
1^st^	221(31.6%)
2^nd^	179(24.9%)
3^rd^	159(22.7%)
> = 4^th^	145(20.7%)

^*^Others^1^ = catholic Judith, Waqefata, Adventist, *Other^2^ = NGO, Job seekers

### Mobile phone ownership by socio-demographic characteristics

Mobile phone ownership varied across different socio-demographic variables. Among 699 respondents, 630(90.1%) had a mobile phone and from these respondents, 361(0.1%) of them had a featured mobile phone. A feature phone is a mobile phone with features such as the ability to store and play music but lacking advanced functionality like a touchscreen interface, internet access, and an operating system that can run downloaded apps [74](Table 2).

**Table 2:** Mobile phone ownership by the socio-demographic characteristic of pregnant women attending ANC at public health facilities in Ambo town west Shoa zone, Ethiopia, 2022G.C (*n* = 699)

Characteristics	Total (%)
Mobile phone ownership	
Yes	630(90.1%)
No	69(9.9%)
Type of mobile phone	
Smartphone	269(44.9%)
Featured phone	361(55.1%)
Duration of Mobile Phone Use in Years	
< = 2	145(23.1%)
3–4	319(50.6%)
> 4	166(26.3%)
Mobile phone sharing use with family	
Yes	72(10.3)
No	627(89.7%)
Mobile network challenge	
Yes	96(15.2%)
No	534(84.8%)
Power outage for a mobile charging	
Yes	72(11.4%)
No	558(88.6%)

### The magnitude of Intention to use a mobile phone to receive mental health support

In this study, 530 (77.3%) with 95%CI (74% to 80.3%) of women intended to use a mobile phone to receive mental health support during the pregnancy period, if offered the opportunity.

### Measurement model assessment

From our study, the value of Cronbach's alpha which has a value above-recommended value ranging from (0.768–0.936), and composite reliability has a value ranging from (0.835–0.963) was used to test internal reliability. These results generally imply that the constructs have strong internal reliability (Table 3).

**Table 3:** Reliability

Constructs	Number of items	Cronbach’s Alpha	Composite reliability
Perceived usefulness	4	0.910	0.876
Perceived ease of use	4	0.936	0.861
Attitude	3	0.886	0.963
Subjective norm	3	0.838	0.948
Perceived trust	3	0.768	0.851
Perceived privacy	3	0.780	0.835
Intention to use	4	0.883	0.922

We also analyzed convergent validity which is the degree to which different attempts to measure the same construct agree. Typically, the average variance extracted (AVE) value of 0.50 or higher indicates that, on average, the construct explains more than half of the variance of its indicators.

It demonstrates how those indicators have a considerable impact on the unidimensionality of latent variables. According to Table 4 below, the factor loading of the constructs measured for the relevant constructions is all over 0.50 and varies from (0.80–0.94), and the AVE of the constructs is between (0.552–0.797). The prerequisites for convergent validity were thus satisfied.

**Table 4:** Convergent validity

Constructs	Items	Factor loadings	AVE
Perceived usefulness	PU1	0.88	0.676
	PU2	0.93	
	PU3	0.84	
	PU4	0.93	
Perceived ease of use	PEOU1	0.92	0.753
	PEOU2	0.90	
	PEOU3	0.92	
	PEOU4	0.88	
Attitude	ATT1	0.93	0.689
	ATT2	0.93	
	ATT3	0.93	
Subjective norm	SN1	0.90	0.576
	SN2	0.88	
	SN3	0.89	
Perceived trust	PT1	0.92	0.552
	PT2	0.87	
	PT3	0.91	
Perceived privacy	PP1	0.80	0.797
	PP2	0.86	
	PP3	0.84	
Intention to use	ITU1	0.93	0.698
	ITU2	0.94	
	ITU3	0.92	
	ITU4	0.93	

Note: *PP* perceived privacy*, PT* perceived trust*, SN* Subjective norm*, PEOU* Perceived ease of use*, PU* Perceived usefulness*, ATT* Attitude*, ITU* Intention to use*, AVE* average variance extracted*, CA* Cronbach's alpha

All diagonal values were greater than matching row and column values, indicating that all measurement variables load more heavily on their respective construct than on the other constructs. Table 5, illustrates the discriminant validity of the constructs, with correlation among constructs and the square root of AVE on the diagonal.

**Table 5:** Discriminant validity

	**ITU**	**PU**	**PEOU**	**ATT**	**SN**	**PT**	**PP**
ITU	**0.836**						
PU	0.667***	**0.822**					
PEOU	0.599***	0.520***	**0.868**				
ATT	0.767***	0.598***	0.671***	**0.830**			
SN	0.151***	0.345***	0.150***	0.134***	**0.759**		
PT	0.637***	0.641***	0.486***	0.634***	0.158***	**0.743**	
PP	0.175***	0.153***	0.164***	0.175***	0.059	0.166***	**0.833**

Note: (***) = In table 5 above the symbol (***) shows that all the diagonal values were greater than matching row and column values, indicating that all measurement variables load more heavily on their respective construct than on the other constructs

A model modification was made, to increase the model fitness by creating covariance of error terms based on the magnitude of modification indices. The model was retested after the changes were made, and sufficient overall goodness of fit values were obtained (Table 6).

**Table 6:** Model fit summary of the research

Matrix indices	cut-off point	Result of this Study	Interpretation
(X^2^/df)	< 3	2.079	Accepted
NFI	> 0.9	0.945	Accepted
GFI	> 0.9	0.9	Accepted
AGFI	> 0.8	0.862	Accepted
CFI	> 0.9	0.958	Accepted
RMSR	< 0.08	0.0346	Accepted
*RMSEA*	< *0.08*	0.066	Accepted

*χ2/df* chi-square divided by degrees of freedom*, RMSEA* Root Mean Square Error of Approximation*, GFI* Goodness of fit index*, AGFI* Adjusted goodness of fit index*, CFI* Comparative fit index*, NFI* Normed fit index*, RMSR* Root mean square of standardized residual

**Fig. 2:**
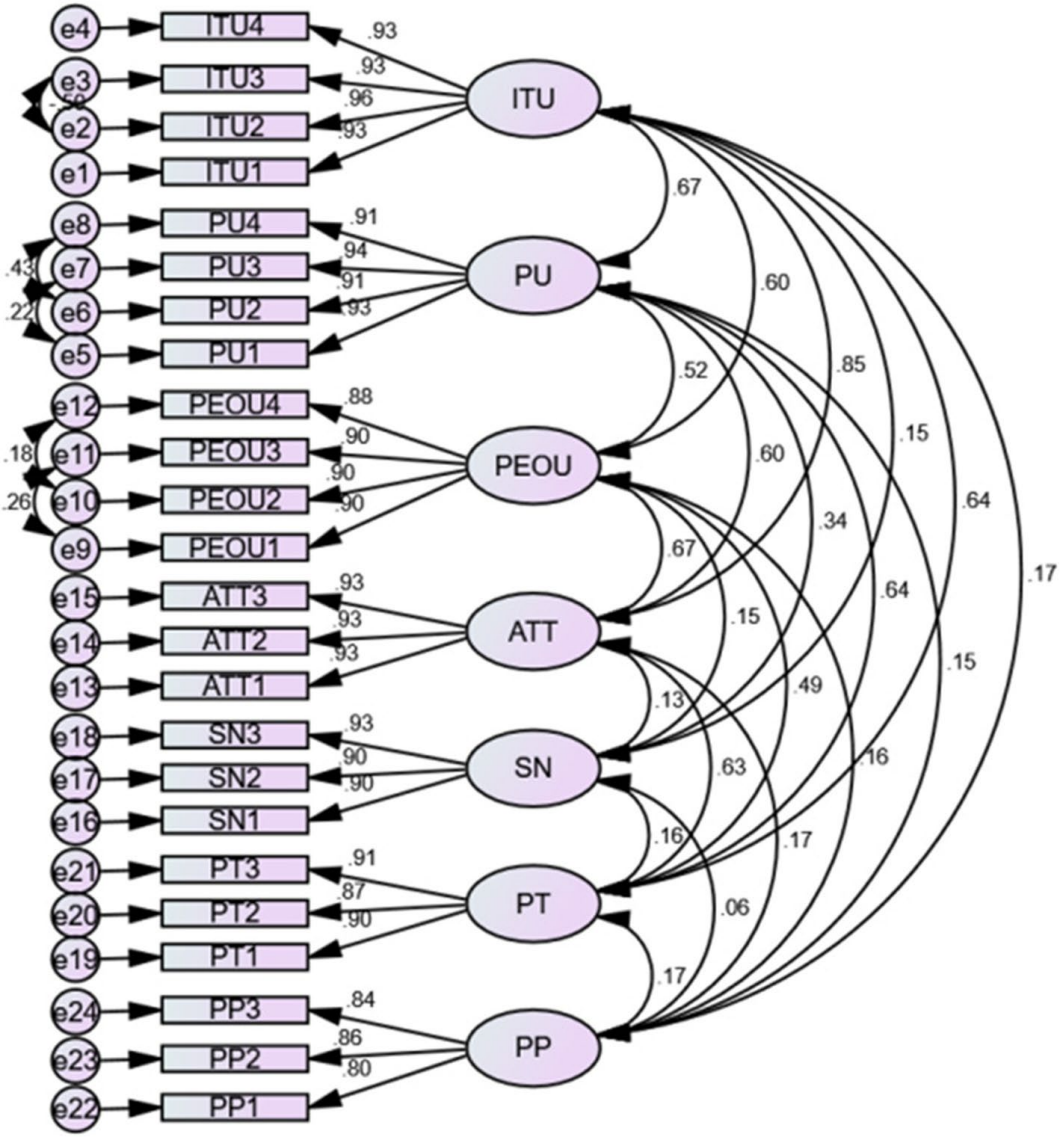
Confirmatory factor analysis of intention to use a mobile phone to receive mental health support among women attending antenatal care at public health facilities in Ambo town, West Shoa zone, Ethiopia 2022. *PP* = *perceived privacy, PT* = *perceived trust, SN* = *Subjective norm, PEOU* = *Perceived ease of use, PU* = *Perceived usefulness, ATT* = *Attitude, ITU* = *Intention to use, e* = *error*

The research model's fitting indices are all above the typical average acceptance threshold, demonstrating that it closely matches the collected data. Diagram illustrating the study's confirmatory factor analysis (Fig. 2).

### Structural model assessment

To determine the relationships between the constructs in the research model, a structural model was developed. The hypotheses were tested using the bootstrap method with a 0.05 level of significance (*p* < 0.05) and standardized path coefficients for the strength of associations. Overall, the structural model fit statistics suggested a satisfactory model fit: GFI = 0.938, AGFI = 0.862, NFI = 0.945, CFI = 0.958, SMSR = 0.0346, RMSEA = 0.066 and x^2^/df = 2.317.

The findings from the measurement model sections have conclusively shown that the data and the model proposed in the current study fit each other quite well. To put it another way, the model is suitable for additional investigation, such as hypothesis testing.

The study finding shows that PU, PT, and attitude had a direct positive and significant effect on pregnant women's intention to use a mobile phone to receive mental health support. H1, H2, H4, H5, H6, H7, H9, and H10 were accepted and verified. H3, H8, and H11, on the other hand, were rejected (Table 7).

**Table 7:** Structural model assessment and Hypothesis test results (*n* = 699)

Parameters	Hypothesis	Estimate	P- *P*-value	Decision
ITU < –-PU	H1	0.228	*** .	Support
ATT < –-PU	H2	0.350	*** .	Support
ITU < –-PEOU	H3	-0.001	0.787	Not support
PU < –-PEOU	H4	0.242	***	Support
ATT < –-PEOU	H5	0.494	***	Support
ITU < –-ATT	H6	0.665	***	Support
PU < –-SN	H7	0.230	***	Support
ITU < –-SN	H8	-0.033	0.149	Not support
ITU < –-PT	H9	0.09	0.005	Support
PU < –-PT	H10	0.493	***	Support
ITU < –-PP	H11	0.016	0.485	Not support

Variables with*** indicate statistically significant at *p*-value < 0.001

*PP* perceived privacy, *PT* perceived trust, *SN* Subjective norm, *PEOU* Perceived ease of use, *PU* Perceived usefulness, *ATT* Attitude, *ITU* Intention to use

Trust has a significant influence on perceived usefulness (β = 0.493, *p* < 0.001) leading to the acceptance of H10, the subjective norm has a positive direct significant influence on perceived usefulness (β = 0.229, *p* < 0.001), and perceived ease of use has a direct positive significant influence on perceived usefulness (β = 0.242, *p* < 0.001) are significant among the hypothesized predictors of perceived usefulness. Among predictors of perceived usefulness trust has the strongest significant association with (β = 0.493).

The path from perceived usefulness has a positive direct significant effect (β = 0.228, *p* < 0.001) on intention to use, attitude toward using to intention to use (β = 0.665, *P* < 0.001), and trust to intention to use (β = 0.09, *p* = 0.005) respectively have a direct positive effect on the intention to use a mobile phone to receive mental health support. Among these predictors which had a direct positive significant effect on the intention to use a mobile phone, attitude has the strongest association with (β = 0.665, *P* < 0.001) on intention to use a mobile phone to receive mental health support.

Perceived ease of use has a positive direct effect on perceived usefulness (β = 0.241, *p* < 0.001) and attitude toward using (β = 0.493, *P* < 0.001). As our finding, perceived ease of use has a strong significant effect on attitude towards using (β = 0.493) than perceived usefulness (β = 0.241). But perceived ease of use has no significant effect on the intention to use (β = -0.001, *p* = 0.987), a mobile phone to receive mental health support.

The attitude towards a mobile phone to receive mental health support was positively affected by perceived usefulness (β = 0.350, *p* < 0.001). It was also found that attitude was significantly affected by perceived ease of use (β = 0.493, *p* < 0.001).

Privacy to intention to use (β = 0.016, *p* = 0.485) and subjective norm to intention to use (β = -0.033, *P* = 0.149), was found to be an insignificant determinant of intention to use a mobile phone to receive mental health support and have no direct effect on the intention to use a mobile phone to receive mental health support.

**Fig. 3:**
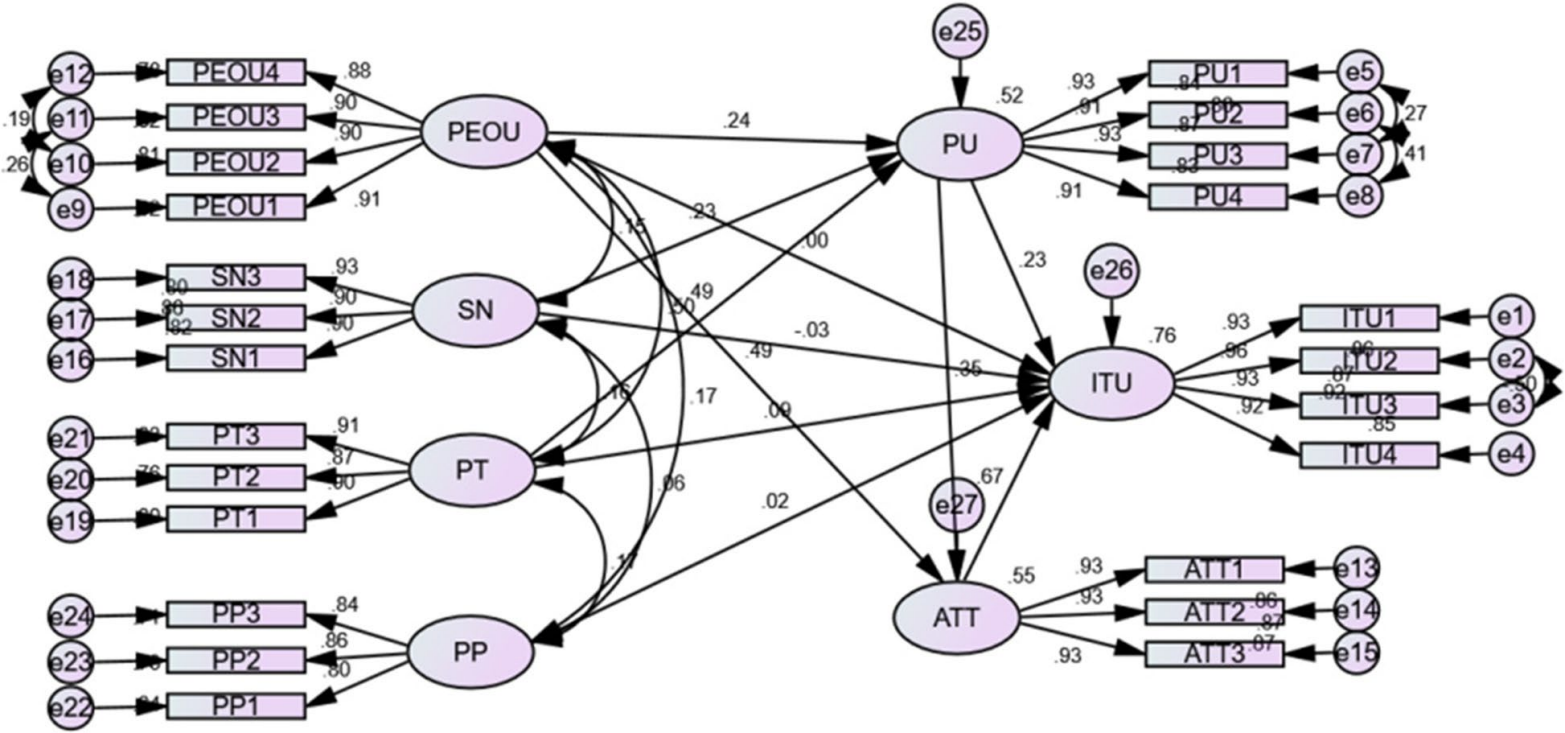
Structural model assessment of intention to use a mobile phone to receive mental health support among women attending antenatal care at public health facilities in Ambo town, West Shoa zone, Ethiopia 2022. *PP* = *perceived privacy, PT* = *perceived trust, SN* = *Subjective norm, PEOU* = *Perceived ease of use, PU* = *Perceived usefulness, ATT* = *Attitude, ITU* = *Intention to use, e* = *error*

For the intention to use construct, the coefficient of determination R^2^ is 0.76. This means that the predictors of intention to use a mobile phone to receive mental health support explain 76% of the variance in intention to use. Subjective norm, trust, and perceived ease of use account for 52% of the variance in perceived usefulness. 55% of the variance in attitude is explained by perceived usefulness and ease of use. As shown in Fig. 3 the result of the structural equation modeling refers to a framework or representation of the relationships between variables (Fig. 3).

### Mediation test

When the predictor first has a substantial impact on the mediator, the mediator then has a significant impact on the criterion variable, and ultimately the predictor has a significant impact on the criterion variable in the absence of the mediator's influence, the situation is said to be in mediation [75]. In this study perceived usefulness has a complete mediation effect of subjective norms on the intention to use a mobile phone to receive mental health support. Perceived usefulness has a complete mediation effect of perceived ease of use on the intention to use a mobile phone to receive mental health support.

Our study showed that an attitude has complete mediation on the path of perceived ease of use to intention to use, and partial mediation on the path of perceived usefulness to intention to use a mobile phone to receive mental health support.

Perceived usefulness also has a partial mediation effect on the path from trust to intention to use. As a result, the relationship between trust and intention to use is partially mediated by perceived usefulness. As shown in Table 8 test results of mediation effect.

**Table 8:** Test results of mediation effect

Hypothesis	Path	Effect	*P*-value	Significant	Kind of mediation	Decision
H12	SN → PU → ITU	Total effect Indirect effect Direct effect	0.021 0.000 0.237	Yes Yes No	Complete mediation	Support
H13	PEOU → PU → ITU	Total effect Indirect effect Direct effect	0.000 0.000 0.984	Yes Yes No	Complete mediation	Support
H14	PU → ATT → ITU	Total effect Indirect effect Direct effect	0.000 0.000 0.000	Yes Yes Yes	Partial mediation	Support
H15	PEOU → ATT → ITU	Total effect Indirect effect Direct effect	0.000 0.000 0.624	Yes Yes No	Complete mediation	Support
H16	Trust → PU → ITU	Total effect Indirect effect Direct effect	0.000 0.000 0.028	Yes Yes Yes	Partial mediation	Support

## Discussion

The proportion of intention to use a mobile phone to receive mental health support among pregnant women in this study (77.3%) is lower than in studies conducted in Nigeria stating that the interest and acceptability of mobile phones to receive mental illness information and care during pregnancy was 97% [76]. The discrepancy may result from different levels of digitalization investment and information and communication technology infrastructure across nations.

This is also lower than the study conducted in India states that the willingness to receive mental health support through mobile phones among perinatal mothers was 96% [77]. This discrepancy might be due to the difference in information and communication technology infrastructure and socioeconomic status among the countries.

However, in this study, the intention to use a mobile phone is higher than in a study done in Bangladesh on the intention to use mobile health for mental health was 73% [36], Saudi Arabia mobile health for mental health at 68% [78], and the United States on intention to use mobile apps to monitor mental disorder symptoms 70.6% [79]. The difference might be due to people in these countries being more concerned about their privacy when they are using a mobile phone to share their mental health information.

The results of this study showed that pregnant women's behavioral intention to use a mobile phone was positively affected by perceived usefulness (β = 0.228). This finding is in line with a study that indicates that perceived usefulness influenced women's intention to seek mental health information through mobile phones in Singapore [80] and Indonesia on the intention to use mobile health [81].

Additionally, perceived usefulness has a significant direct positive effect on pregnant women's attitude towards using (β = 0.350) a mobile phone to receive mental health support. This finding is in line with the study conducted in Turkey on the intention to use mobile health [82] and in South Korea on the intention to use a smartphone [83]. Pregnant women's attitudes toward use were more likely to be influenced, as they believe that mobile phones improve their mental health access and health status.

Our findings show that perceived ease of use had not directly affected behavioral intention to use (β = -0.001) a mobile phone to receive mental health support during the pregnancy period. This finding is consistent with another study in Bangladesh [84], and China on the intention to use e-consultation [85]. The result of this is contrary to studies done on the Intention to use e-health in Taiwan [40], and Malaysia [41]. The other possible explanation might be because of the popularity of mobile phones in the study area, it is reasonable to believe that pregnant women can easily learn how to use a mobile phone to receive mental health support; thus, perceived ease of use has no longer plays a role in the promotion.

In our finding perceived ease of use has a strong positive impact on perceived usefulness (β = 0.241). This result is consistent with a study conducted in Bangladesh on the intention to use mobile health [86]. If users think that using a mobile phone to receive mental health support is simple to use, it will affect the women's ability to judge whether it is helpful [86]. According to the findings of this particular study, it can be deduced that the women's interface's friendliness can have a significant impact on a woman's commitment to using a mobile phone system to receive mental health support. So this shows that when women think that using a mobile phone is simple to use they will be more inspired to use it.

Likewise, perceived ease of use has a positive significant effect on the attitude of pregnant women towards using (β = 0.494) a mobile phone to receive mental health support. This finding is in line with the study conducted on the intention to use mobile health in Spain [87]. This shows that if pregnant women perceive that the use of a mobile phone is easy they will develop a positive attitude towards using it.

Pregnant women’s attitudes had a direct positive effect on their intention to use (β = 0.665) a mobile phone to receive mental health support. This finding is consistent with other findings in China that were conducted intention of using online mental health intervention among women [86]. It can be inferred that promoting a positive attitude toward mobile phone use can enhance users' intention to use a mobile phone to receive mental health support [86]. As a result, promotional activities should be launched to elucidate the attitudes of individuals and educate them about the availability of mobile phone-based mental health support and interventions, with a focus on groups most at risk for mental health problems [86].

We can conclude that when women have a favorable attitude toward using their mobile phones to receive mental health support, the more they intend to use a mobile phone to receive mental health support.

In addition to the fundamental TAM model, the current study also examined trust, subjective norms, and privacy. Numerous studies have demonstrated the importance of these variables in the uptake of e-services including e-commerce, e-health, and e-government [88, 89].

Subjective norms had a positive direct effect on the perceived usefulness (β = 0.23) of mobile phones to receive mental health support. This is in line with studies conducted in India on the intention to use mobile health [44], China [45], the Arab world on the intention to use a smartphone [90], and Germany on the intention to use mobile mental health [10]. This shows that when women believe the system is beneficial and their friends, family, and important people push them to use it they can think that it is useful.

According to this study, subjective norms had no direct positive and significant effect on the intention to use (β = -0.033) a mobile phone. This result is consistent with different studies in India on the intention to use mobile health [33, 44]. This may be because pregnant women may not perceive external pressure to use a new system from their family, husband, religious leader, or other important people in their lives. This finding contradicts Venkatesh and Davis' [43], claim that SN can directly influence user acceptance. This distinction might be due to the unique characteristics of mobile phone users. When target users are less influenced by social norms and have a weaker desire to belong to a specific social group subjective norm has no role in influencing intention to use [45].

In this study, trust has a direct positive and significant effect on the intention to use (β = 0.09) and perceived usefulness (β = 0.493) of a mobile phone to receive mental health support. This means that higher levels of trust are associated with higher levels of perceived usefulness and behavioral intention to use. This is consistence with studies in Germany on the intention to use mobile mental health [10] and England on the intention to use mobile health applications [35]. Users will be less hesitant to use mobile phone services if they begin to think that these services are reliable and trustworthy [10]. This shows that women’s trust increases their confidence in their mobile phones and healthcare providers to acquire services for mental health support to obtain better health services in the future.

Privacy has no direct positive significant effect on the intention to use (β = 0.016) a mobile phone to receive mental health support. This finding is consistent with studies in Egypt on the intention to mobile health [35], Bangladesh on the intention to use e-health [46], and China on the intention to mobile health [52]. However, contrary studies conducted in Pakistan on telemedicine [48], China on the intention to use telehealth [53], and Germany on privacy concerns on intention to use PHR [54], indicated that Perceived Privacy had a positive effect on the intention to use. The difference might reflect the fact that people in the study area are not overly concerned with privacy and disclosing information to third parties.

According to this study, attitude towards using has the strongest effect on the intention to use followed by perceived usefulness and trust which is a highly significant effect as compared to the other construct related to intention to use a mobile phone to receive mental health support. Therefore, these constructs were the potential predictors of intention to use a mobile phone. Attitude toward using a mobile phone was the strongest predictor of intention to use in line with a study in Ethiopia on electronic health adoption [37].

Regarding mediation, an attitude has complete mediation on the path of intention to use and perceived ease of use and partial mediation on the path of intention to use and perceived usefulness. This result is consistent with the study [53]. Perceived usefulness also has a partial mediation effect on the path of intention to use and trust. This is in line with the study [53]. This finding shows that increasing the system's trustworthiness primarily increases women's perceived usefulness and increases their intention to use a mobile phone to receive mental health support.

Attitude plays a complete mediating role between perceived ease of use and intention to use a mobile phone to receive mental health support among pregnant women. This result is in line with findings that state that attitude had a mediating effect between perceived usefulness, perceived ease of use, and pregnant women's intentions to use a mobile phone [53, 55].

This suggests that attitude serves as a crucial bridge in this process. Indicating that attitude had complete mediation and perceived ease of use can only change the intention to use a mobile phone of pregnant women by altering their acceptance of mobile phone service.

The positive effect and the significant results of the perceived usefulness through direct, indirect, and total effects on intention to use through attitude suggest that attitude serves as a partial mediator between perceived usefulness and intention to use. This finding is consistent with [55]. This shows that if the perception of using mobile phones to receive mental health support is useful then they develop favorable attitudes toward using a mobile phone to receive mental health support increasing women’s intention to use the services.

## Limitations of the study

There are some limitations to this study. First, since the study was an institution-based cross-sectional survey a result, may not show a significant cause-effect relationship.

Second, only respondents who came for the ANC visit were interviewed, and not included those who did not come to the institution.

Third, this study did not include the private health facility that existed in the study area.

Fifth the way intention to use is operationalized as intended or unintended based on the median score may bias our results. To mitigate the limitation of the sampling strategy in the study, the best approach would be to ensure that the sample frame is complete, current, and accurately represents the target population.

## Conclusion

In this study, we found that pregnant women have a high intention to use a mobile phone to receive mental health support during the pregnancy period.

According to our findings, constructs like perceived usefulness, trust, and attitude have a direct positive and significant effect on the intention to use a mobile phone to receive mental health support among prenatal women. Moreover, the most predictive factor for the intention to use a mobile phone to receive mental health support among pregnant women was revealed to be the attitude toward using a mobile phone. The second most predictive factor was perceived usefulness followed by trust was also discovered to be the major determinant of an intention to use a mobile phone.

An attitude has complete mediation on the path of intention to use and perceived ease of use and partial mediation on the path of intention to use and perceived usefulness and perceived usefulness act as a complete mediator between perceived ease of use intention to use subjective norms, and intention to use and partial mediator between trust and intention to use a mobile phone to receive mental health support.

The findings demonstrate the durability of the modified TAM model in predicting pregnant women's intention to use a mobile phone to receive mental health support as well as its strong explanatory power of R^2^ 76%. Furthermore, by incorporating subjective norms, trust, and privacy as new factors in the setting of Ethiopian pregnant women of intention to use a mobile phone to receive mental health support, the current study expanded and supported the preexisting TAM model.

### Supplementary Information

**Additional file 1: Supplementary file 1. **The proposed hypotheses of the model in the study.** Supplementary file 2. **Questionnaires.

## Data Availability

The corresponding author will make the datasets created and/or used for this study reasonably available upon request.

## Abbreviations

AVE: Average Variance Extracted
AMOS: Analysis Of A Moment Structure
ANC: Antenatal Care
CMHS: College Of Medicine And Health Science
DHS: Demographic Health Survey
EDHS: Ethiopian Demographic Health Survey
ITU: Intention To Use
LMICS: Lower Middle-Income Countries
PEOU: Perceived Ease Of Use
PU: Perceived Usefulness
SEM: Structural Equation Modeling
SN: Subjective Norm
SPSS: Statistical Package Of Social Science
TAM: Technology Acceptance Model
TPB: Theory Of Plan And Behavior
TRA: Theory Of Reason And Action
WHO: World Health Organization
