# Supplementary material for: Intention to use a mobile phone to receive mental health support and its predicting factors among women attending antenatal care at public health facilities in Ambo town, West Shoa zone, Ethiopia 2022
Source: BMC Health Serv Res. 2023 Dec 6;23:1368. doi: 10.1186/s12913-023-10392-z (PMC10701993; doi:10.1186/s12913-023-10392-z)
Supplement: Supplementary file 1 — Additional file 1: Supplementary file 1. The proposed hypotheses of the model in the study. Supplementary file 2. Questionnaires. [file 12913_2023_10392_MOESM1_ESM.docx]

**Supplementary file 1**

| **The proposed hypotheses of the model in the study** | |
| --- | --- |
| **Perceive usefulness** | **H1**: Perceived Usefulness will have a positive effect on the intention to use a mobile phone to receive mental health support among pregnant women.  **H2:** Perceived usefulness will have a positive effect on pregnant women’s attitudes towards using a mobile phone to receive mental health support. |
| **Perceived ease of use** | **H3:** Perceived ease of use will have a positive effect on the intention to use a mobile phone to receive mental health support.  **H4:** Perceived ease of use will have a positive effect on the perceived usefulness of using a mobile phone to receive mental health support.  **H5:** Perceived ease of use will have a positive effect on pregnant women’s attitude towards using a mobile phone to receive mental health support. |
| **Attitude** | **H6:** Pregnant women’s attitude towards a mobile phone to receive mental health support will have a positive effect on the intention to use a mobile phone. |
| **Subjective norm** | **H7:** Subjective norms will have a positive effect on the perceived usefulness of mobile phones to receive mental health support among pregnant women.  **H8:** Subjective norms will have a positive effect on the intention to use mobile phones to receive mental health support among pregnant women. |
| **Perceived trust** | **H9:** Perceived Trust will have a positive effect on the intention to use a mobile phone to receive mental health support among pregnant women.  **H10:** Perceived Trust will have a positive effect on the perceived usefulness to receive mental health support among pregnant women. |
| **Perceived privacy** | **H11:** Perceived Privacy will have a positive effect on the intention to use a mobile phone to receive mental health support among pregnant women |
| **Mediation effects** | **H12:** PU mediates the relationship between SN and the Intention to use a mobile phone to receive mental health support.  **H13:** PU mediates the relationship between PEOU and Intention to use a mobile phone to receive mental health support.  **H14:** Pregnant women’s attitude toward using a mobile phone to receive mental health support mediates the relationship between PU and Intention to use.  **H15:** Pregnant women’s attitude toward using a mobile phone to receive mental health support mediates the relationship between PEOU and Intention to use.  **H16:** PU mediates the relationship between Trust and Intention to use a mobile phone to receive mental health support. |

**Supplementary file 2 Questionnaires**

**Part I: Questionnaires related to sociodemography of characteristics of eligible mothers.**

| **Code** | **Questions** | **Response** | | |
| --- | --- | --- | --- | --- |
| S1 | Age in present years | ----------- | | |
| S2 | Marital Status | 1. Married 2. Single 3. Divorced  4. Separated 5. Widowed | | |
| S3 | Religion | 1) Orthodox 2) Muslim 3) Protestant 4) Catholic 5) Others(specify) | | |
| S4 | Respondent’s educational background | 1. No formal Education  2. Primary Education(1-8)  3. Secondary Education(9-12)  4. Above Secondary | | |
| S5 | Respondent’s occupation | 1) Student  2) Government employer  3) Merchant  4) Farmer  5) Housemaid  6) Job seeker/jobless  7) Others(specify) | | |
| S6 | Residence | 1. Urban 2. Rural | | |
| S7 | Distance to health facility(in minutes) | ---------------Minutes | | |
| S8 | ANC visit number? | 1) 1^st^ 2) 2^nd^ 3) 3^rd^ 4) 4^th^ | | |
| S9 | Number of birth | ------------------ | | |
|  | |  | | |
| **Part II: Mobile related Questionnaires** | |  |  |  |
| **Code** | **Questions** | | **Response** | |
| MD1 | Currently, have a mobile phone? | | 1)Yes 2)No |  |
| MD2 | If yes for MD1 for how long have you been using a mobile phone in years? | | -------------- | If No skip to MD6 |
| MD3 | What types of mobile phones are you currently using? | | 1) Smartphone  2) featured phone |  |
| MD4 | Have you considered facing mobile network challenges? | | 1) Yes 2) No |  |
| MD5 | Is a mobile phone charged due to a power outage? | | 1) Yes 2) No |  |
| MD6 | The phone is shared with others in the house? | | 1. Yes 2.No |  |

**Part III: Questionnaires related to modified technology of acceptance model**

**Please select the one to which your answer is exactly matching of the given number to show your answer. Strongly disagree=1 Disagree=2 Neutral=3 Agree=4 strongly agree=5**

| **S/No** | | **Items** | **Questions** | | **Response** | | | | | | | |
| --- | --- | --- | --- | --- | --- | --- | --- | --- | --- | --- | --- | --- |
|  |  |  |  |  | **1** | | **2** | | **3** | **4** | | **5** |
| **Perceived usefulness** | | | | |  |  | | |  |  | |  |
| 1 | | PU1 | Using a mobile phone for mental health support would improve the quality of my mental health care. | | 1 | | 2 | | 3 | 4 | | 5 |
| 2 | | PU2 | Using a mobile phone to receive mental health support would improve my access to mental health care services. | | 1 | | 2 | | 3 | 4 | | 5 |
| 3 | | PU3 | Using a mobile phone to receive mental health support would be useful in my daily health care. | | 1 | | 2 | | 3 | 4 | | 5 |
| 4 | | PU4 | I think that using a mobile phone to receive mental health support would provide me with very useful services. | | 1 | | 2 | | 3 | 4 | | 5 |
| **Perceived ease of use** | | | | | | | | | | | | |
| 5 | PEOU1 | | | I would think using a mobile phone would not be very difficult for me to receive mental health support. | 1 | | 2 | | 3 | | 4 | 5 |
| 6 | PEOU2 | | | I would think it is easy for me to interact with health care providers using a mobile phone to receive mental health support. | 1 | | 2 | | 3 | | 4 | 5 |
| 7 | PEOU3 | | | Interacting with a mobile phone would be clear and understandable for me to receive mental health support from health care providers. | 1 | | 2 | | 3 | | 4 | 5 |
| 8 | PEO4 | | | Using a mobile phone to receive mental health support will not be complicated | 1 | | 2 | | 3 | | 4 | 5 |
| **Attitude** | | | | | | | | | | | | |
| 9 | | AT1 | Using a mobile phone for receiving mental health support would be a very good idea. | | 1 | | 2 | | 3 | | 4 | 5 |
| 10 | | AT2 | In my opinion, it would be very desirable for me to use a mobile phone to receive mental health support. | | 1 | | 2 | | 3 | | 4 | 5 |
| 11 | | AT3 | It would be much better to use a mobile phone to receive mental health support. | | 1 | | 2 | | 3 | | 4 | 5 |
| 12 | | AT4 | I like using mobile phones to receive mental health support. | | 1 | | 2 | | 3 | | 4 | 5 |
| **Subjective norm** | | | | | | | | | | | | |
| 13 | | SN1 | People who influence my behavior think that I should use a mobile phone to receive mental health support. | | 1 | | 2 | | 3 | | 4 | 5 |
| 14 | | SN2 | People who are important to me think that I should use a mobile phone to receive mental health support. | | 1 | | 2 | | 3 | | 4 | 5 |
| 15 | | SN3 | People whose opinions I value think I should use a mobile phone to receive mental health support. | | 1 | | 2 | | 3 | | 4 | 5 |
| **Perceived trust** | | | | | | | | | | | | |
| 16 | | PT1 | Mobile phone services would be trustworthy to receive mental health support. | | 1 | | | 2 | 3 | | 4 | 5 |
| 17 | | PT2 | Mobile phone systems will require me to be cautious with this technology to get support for mental health. | | 1 | | | 2 | 3 | | 4 | 5 |
| 18 | | PT3 | I feel satisfied and confident that I will be able to rely on the benefits of mobile phone services to receive mental health support. | | 1 | | | 2 | 3 | | 4 | 5 |
| **Perceived privacy** | | | | | | | | | | | | |
| 19 | | PP1 | Others might not take control of my information if I used mobile phone services to receive mental health support. | | 1 | | | 2 | 3 | | 4 | 5 |
| 20 | | PP2 | Signing up for and using mobile phone services would not lead to a loss of privacy for me because my personal information could be used without my knowledge to receive mental health support. | | 1 | | | 2 | 3 | | 4 | 5 |
| 21 | | PP3 | My use of mobile phone services would not cause me to lose control over the privacy of my information to receive mental health support. | | 1 | | | 2 | 3 | | 4 | 5 |
| **Intention to use** | | | | | | | | | | | | |
| 22 | | IU1 | I intend to use a mobile phone to receive mental health support. | | 1 | | | 2 | 3 | | 4 | 5 |
| 23 | | IU2 | I intend on informing my relatives and friends about the mobile phone service to receive mental health support. | | 1 | | | 2 | 3 | | 4 | 5 |
| 24 | | IU3 | Given that I have access to the mobile phone for mental health support, I predict that I would use it. | | 1 | | | 2 | 3 | | 4 | 5 |
| 25 | | IU4 | Whenever I would need remote mental health support from professionals, I would gladly use a mobile phone service to get mental health support. | | 1 | | | 2 | 3 | | 4 | 5 |
